# Copper supported Dowex50WX8 resin utilized for the elimination of ammonia and its sustainable application for the degradation of dyes in wastewater

**DOI:** 10.1038/s41598-024-69839-w

**Published:** 2024-08-27

**Authors:** Mohamed M. Khamis, Abeer S. Elsherbiny, Ibrahim A. Salem, Marwa A. El-Ghobashy

**Affiliations:** Chemistry Department, Faculty of Science, Tanta University, Tanta, 31527 Egypt

**Keywords:** Ammonia, Dowex-50WX8, Complexation, D-Cu(II)-ammine composite, Dyes, Degradation, Environmental sciences, Chemistry

## Abstract

To obtain high efficient elimination of ammonia (NH_4_^+^) from wastewater, Cu(II), Ni(II), and Co(II)) were loaded on Dowex-50WX8 resin (D-H) and studied their removal efficiency towards NH_4_^+^ from aqueous solutions. The adsorption capacity of Cu(II)-loaded on D-H (D-Cu^2+^) towards NH_4_^+^ (q_e_ = 95.58 mg/g) was the highest one compared with that of D-Ni^2+^ (q_e_ = 57.29 mg/g) and D-Co^2+^ (q_e_ = 43.43 mg/g). Detailed studies focused on the removal of NH_4_^+^ utilizing D-Cu^2+^ were accomplished under various experimental conditions. The pseudo-second-order kinetic model fitted well the adsorption data of NH_4_^+^ on D-Cu^2+^. The non-linear Langmuir model was the best model for the adsorption process, producing a maximum equilibrium adsorption capacity (q_max_ = 280.9 mg/g) at pH = 8.4, and 303 K in less than 20 min. The adsorption of NH_4_^+^ onto D-Cu^2+^ was an exothermic and spontaneous process. In a sustainable step, the resulting D-Cu(II)-ammine composite from the NH_4_^+^ adsorption process displayed excellent catalytic activity for the degradation of aniline blue (AB) and methyl violet 2B (MV 2B) dyes utilizing H_2_O_2_ as an eco-friendly oxidant.

## Introduction

Intensive agricultural development, massive industrial expansion, and rapid population growth have always been associated with adverse environmental effects, especially on water^1^. Water pollution has become one of the most serious problems facing humans and living organisms globally and makes it difficult to ensure sufficient and potable water^2^. When pollutants present in industrial effluents enter the ecosystem, they pose a major hazard to the environment^3,4^. Among these pollutants, ammonia (NH_4_^+^) is utilized in the production of numerous nitrogenous products, 83% of which serve as agricultural fertilizers^5^. Another hazardous pollutant is the organic dyes that are used in the printing inks, cosmetics, paper, textiles, and pharmaceutical industries^6^.

Although NH_4_^+^ is a necessary nutrient for plant metabolism, excessive levels of it enter the water stream through leaching and surface runoff, leading to a high risk of water eutrophication. This water eutrophication has adverse impacts on the environment and aquatic life^7^. Additionally, NH_4_^+^ can be transformed into carcinogens such as nitrate (NO_3_^−^) and nitrite (NO_2_^−^), which could be dangerous to public health and cause human diseases such as methemoglobinemia^8^. As a result, NH_4_^+^ removal from water is mandated by regulatory bodies in many regions. Furthermore, the European Environment Agency (EEA) advised researchers to investigate new methods for NH_4_^+^ removal. Many technologies were developed to eliminate NH_4_^+^ from wastewater, including air stripping^9^, membrane filtration^10^, the flow electrode capacitive deionization (FCDI)^11^, ion exchange^12^, adsorption method^13^, breakpoint chlorination^14^, struvite precipitation ^8^, electrochemical oxidation ^15^, photocatalysis ^16^, nitrification and denitrification ^17^, and anaerobic NH_4_^+^ oxidation ^18^. Among these techniques, the adsorption process is considered a better technology for recovering NH_4_^+^ from wastewater due to its economic and sustainable viability for the environment. Several types of NH_4_^+^ adsorbents were applied, such as natural and synthetic kinds of zeolites ^19^, clay minerals ^20^, carbon-based materials ^21^, polymeric exchange resins ^22^, and polymer hydrogel ^23^. Ion exchange resins have gained significant interest and have become increasingly popular adsorbents for removing NH_4_^+^ from wastewater due to their low cost and simplicity of recycling ^24^. However, the selectivity of ion exchange materials is limited by variations in ionic valence, hydration radius, and hydrated energy ^25^. Therefore, in the presence of competing cations, conventional ion exchange materials lack the ability to remove NH_4_^+^ with high selectivity. NH_4_^+^ can be removed through two processes; ion exchange and coordinative complexation, but the most widely utilized selective method is via ligand exchange technology. The selective ligand exchange of NH_4_^+^ by using polymeric cation exchange resins loaded with transition metal cations was suggested to overcome the limitations of selectivity for ion exchange resin ^26,27^. Helfrich proposed the concept of ligand exchange in 1962, while studying the selectivity of ion exchangers for NH_4_^+^ adsorption. According to ligand exchange, it has also been shown that metal-loaded ion exchangers have the ability to adsorb NH_4_^+^ ions from wastewater ^25^. NH_4_^+^ removal through ligand exchange reaction is expressed as (Eq. 1).

1 $${\text{D}}_{2}{\left[\text{Cu}{\left({\text{H}}_{2}\text{O}\right)}_{4}\right]}^{2+} + 4{\text{NH}}_{3\left(\text{aq}\right)} \leftrightharpoons {\text{D}}_{2}{\left[\text{Cu}{\left({\text{NH}}_{3}\right)}_{4}\right]}^{2+} + 4{\text{H}}_{2}\text{O }$$

where, D denotes the strong acidic resin, such as Dowex-50WX8 (D-H). Pehlivan and Altun investigated the ion exchange of various cations such as Cd^2+^, Cu^2+^, Zn^2+^, Ni^2+^, and Pb^2+^ from aqueous solutions with Dowex 50W synthetic resin^28^. The atomic number, valency, and degree of ionization of the exchanged metals significantly impact the –SO_3_H group's selectivity. Previous studies showed that D-H was used in a batch adsorption study to remove NH_4_^+^ from real and synthetic wastewater^29^. The authors found that 3 g of D-H exhibited a removal efficiency of 89.4% within 5 min using the real wastewater at an initial NH_4_^+^ concentration of 22.7 mg/L. Dowex-50WX4 in Na^+^ form was used to eliminate NH_4_^+^ from water containing low concentrations of NH_4_^+^ using the ion exchange method. The maximum quantity of NH_4_^+^ uptake was 4.77 mg/g within the equilibrium time of 20 min at 30 °C^30^. The ligand exchange concept led to the loading of many transition metals onto the sulfonated polymeric resin, such as Amberlite IR-120, and was used for removing NH_4_^+^ from polluted wastewater^31^. A metal ion links to a polymeric exchange resin via electrostatic attraction, whereas NH_4_^+^ is complexed with the metal ions on the ion exchange resin through ligand exchange with aqua molecules in the metal solvation shell. To our knowledge, no reports have explored utilizing D-Cu^2+^, D-Ni^2+^, and D-Co^2+^ for the elimination of NH_4_^+^ from wastewater based on the ligand exchange technique. This study demonstrates the high removal efficiency of NH_4_^+^ (low concentrations) using a small amount of D-Cu^2+^ in only 20 min which is better than our previous work^32^. As well as this study reveals the ability to use solid waste as a low-cost catalyst for the enhancement of the elimination of dyes from wastewater. So, the removal of NH_4_^+^ from wastewater by ligand exchange technique was the main goal of this study. To achieve this goal D-H resin was successively loaded with three different cations namely, Cu(II), Ni(II), and Co(II) through the impregnation method to create D-Cu^2+^, D-Ni^2+^, and D-Co^2+^, respectively. These adsorbents were used to remove NH_4_^+^ from an aqueous solution. The ideal conditions for removing NH_4_^+^ from wastewater using D-Cu^2+^ were determined by evaluating the operating variables, such as contact time, pH, initial concentration, temperature, and coexisting ions. A set of experiments was carried out to illustrate the kinetics, isotherm models, and thermodynamics of NH_4_^+^ adsorption from aqueous solution using D-Cu^2+^. Additionally, the resulting product, D-Cu(II)-ammine composite, demonstrated high catalytic activity for the degradation of some organic dyes in wastewater. Therefore, in a sustainable step the resulting product, D-Cu(II)-ammine composite, was applied as a catalyst for the degradation of aniline blue and Methyl violet 2B in the presence of hydrogen peroxide as an eco-friendly oxidant.

## Results and discussion

### Loading of metal cations on D-H

D-H is a popular cation exchanger for removing metal ions from water because it has sulfonate functional groups for binding metal ions to it. ICP-OES measurements revealed that D-H was loaded with different concentrations of Cu(II), Ni(II), and Co(II). The loaded amount of these metals on 1 g of D-H was 296 mg/g, 90 mg/g, and 60 mg/g, respectively.

### Characterization

#### FT-IR

**Figure 1:**
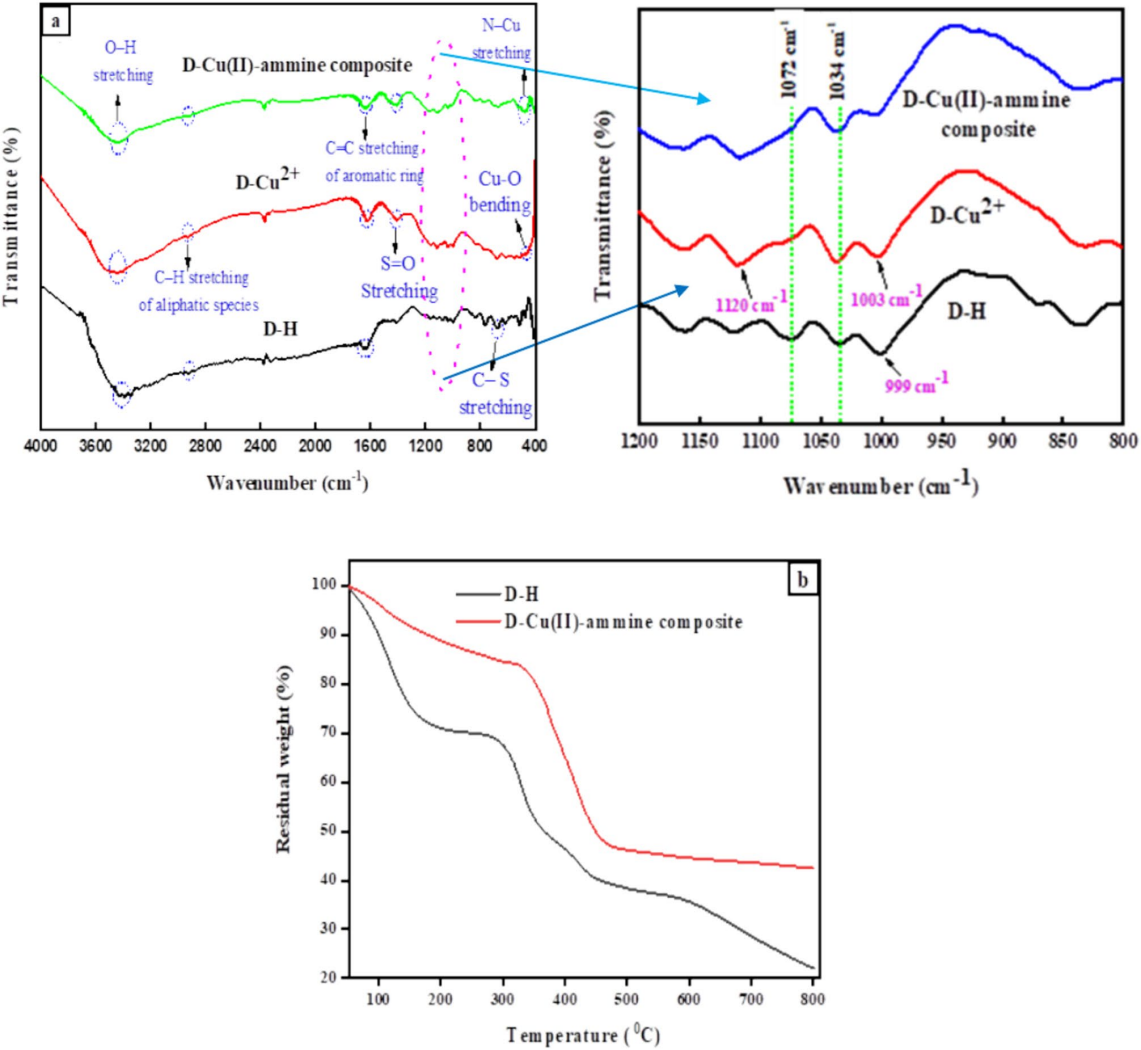
(**a**) FT-IR spectra of D-H, D-Cu^2+^, and D-Cu(II)-ammine composite, (**b**) TGA curves of D-H and D-Cu(II)-ammine composite.

Figure 1a displays the FT-IR spectra of D-H, D-Cu^2+^, and D-Cu^2+^ after NH_4_^+^ adsorption (D-Cu(II)-ammine composite). The spectrum of pure D-H displayed an absorption peak at 3420 cm^−1^, which may be interpreted for the stretching OH vibrations of physically adsorbed water molecules. The broad peak of C–H stretching in aliphatic species (C–H and –CH_2_ groups) appeared at 2925 cm^−1^, while the band at 1640 cm^−1^ refers to the aromatic ring of C=C stretching vibration^33^. The stretching vibration of S=O in the sulphonic acid group is related to the band at 1420 cm^−1^. The bands at 1034 cm^−1^ and 1072 cm^−1^ are due to the symmetric and antisymmetric stretching vibrations of SO_3_ group, respectively^34^. The band at 836 cm^−1^ showed a bending vibration of C–H out of the plane of the aromatic ring, and the band at 664 cm^−1^ was related to C**–**S stretching vibration^35^. FT-IR spectrum of D-H after loading with copper ions indicates that D-Cu^2+^ was formed through hydrogen ion of the sulfonate group which is the exchangeable moiety with copper (II) ions^36^. This leads to the movement of the stretching vibration band of the hydroxyl group from 3420 to 3444 cm^−1^. The two bands at 1072 cm^−1^ and 999 cm^−1^ were moved to 1120 cm^−1^ and 1003 cm^−1^, respectively, indicating the development of the coordinating bond between Cu(II) and the SO_3_ groups of D-H. While, the peak at 664 cm^−1^ remained at the same position as in Fig. 1a of D-H, showing that some sulfonated groups of D-H resin remain free^31^. In addition, the spectrum of D-Cu^2+^ showed a new band at 445 cm^−1^ associated with Cu–O bending^33^. After the adsorption of NH_4_^+^ on D-Cu^2+^, the IR spectrum (Fig. 1a) indicates ammonia bound to the Cu(II) ions, which is confirmed by the blue shift of the stretching band at 1410 cm^−1^ of the SO_2_ group to 1425 cm^−1^. Additionally, a new band at 480 cm^−1^ was attributed to the stretching vibration of N–Cu, confirming that a complex had formed between the Cu(II) ion and the ammonia molecules^31^.

#### TGA

The pristine D-H and the prepared D-Cu(II)-ammine composite exhibit three distinct weight loss stages, according to the TGA thermogram in Fig. 1b. For the raw D-H resin, the first stage results in a weight decrease of about 15.86% at (50–118 °C) temperature range. This is mostly caused by the physical adsorption of water from the surface of D-H. At the temperature range of 118–329 °C, about 24.64% of the weight loss occurs during the second stage, which refers to the decomposition of sulfonated functional groups^37^. The third step in weight loss above 330 °C is the result of polymer backbone decomposition^38^. While the TGA of the D-Cu(II)-ammine composite showed a cumulative weight loss of approximately 55.72% during various steps. At temperatures between 50 and 109 °C, the first step revealed around 5.71% due to the dehydration of adsorbed water molecules. The second stage displayed the complete decomposition of amino ligands of the Cu(II)-ammine complex, with a weight loss of 10.99% at temperatures between 109 and 310 °C^31^. Above 310 °C, there was a 39.02% weight loss in the last stage of the degradation process. This is associated with the organic polymer degradation of D-H, leaving behind a thermally stable metal oxide. The D-Cu(II)-ammine composite has higher thermal stability than the D-H resin^39^.

#### SEM

**Figure 2:**
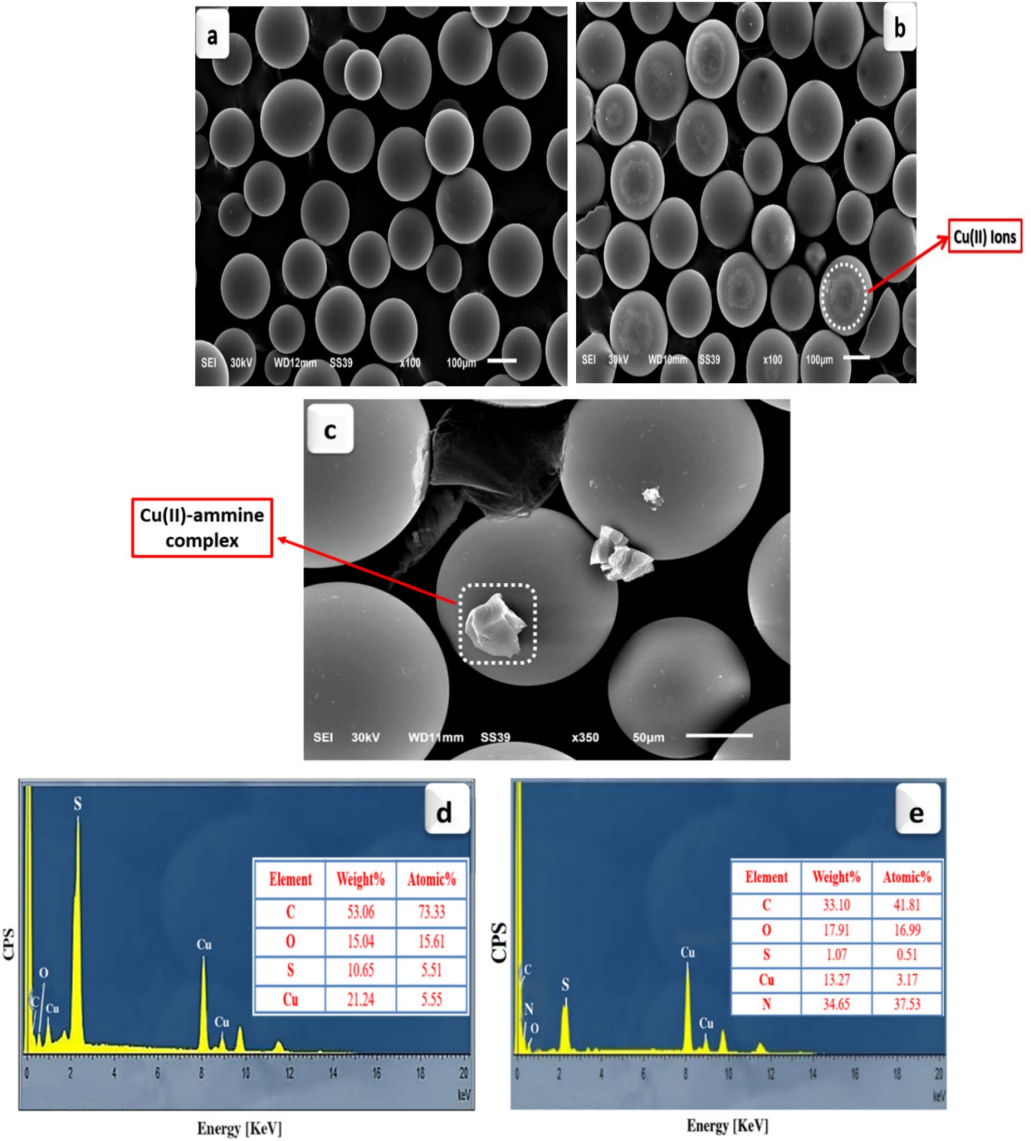
SEM micrographs of (**a**) D-H, (**b**) D-Cu^2+^, (**c**) D-Cu(II)-ammine composite, EDX images of D-Cu^2+^: (**d**) before and (**e**) after the NH_4_^+^ adsorption.

SEM analysis was used to study the surface morphologies of D-H, D-Cu^2+^, and D-Cu(II)-ammine, as shown in Fig. 2a–c, respectively. SEM image of D-H showed randomly distributed spherical shapes with a smooth surface^40^. It was found that the surface of D-Cu^2+^ had covered spherical spots of irregular size (Fig. 2b), confirming that D-H had been impregnated with Cu(II) ions^41^. The surface of D-Cu(II)-ammine composite illustrated the appearance of some bright aggregate particles on the D-Cu^2+^ surface, which demonstrates the adsorption of NH_4_^+^ on the surface of D-Cu^2+^.

#### EDX

Figure 2d,e shows EDX spectra of the D-Cu^2+^ before and after the NH_4_^+^ adsorption. The elemental composition of the D-Cu^2+^ is C, O, S, and Cu, which demonstrates the loading of copper ions on the surface of D-H resin (Fig. 2d). A new peak for N was observed after the adsorption process, Fig. 2e, and the ratio of N is about 37.53%. These results confirm the adsorption of NH_4_^+^ onto the D-Cu^2+^ surface.

### Kinetics of removing ammonia using metal supported on Dowex-50WX8 (D-M^n+^)

**Figure 3:**
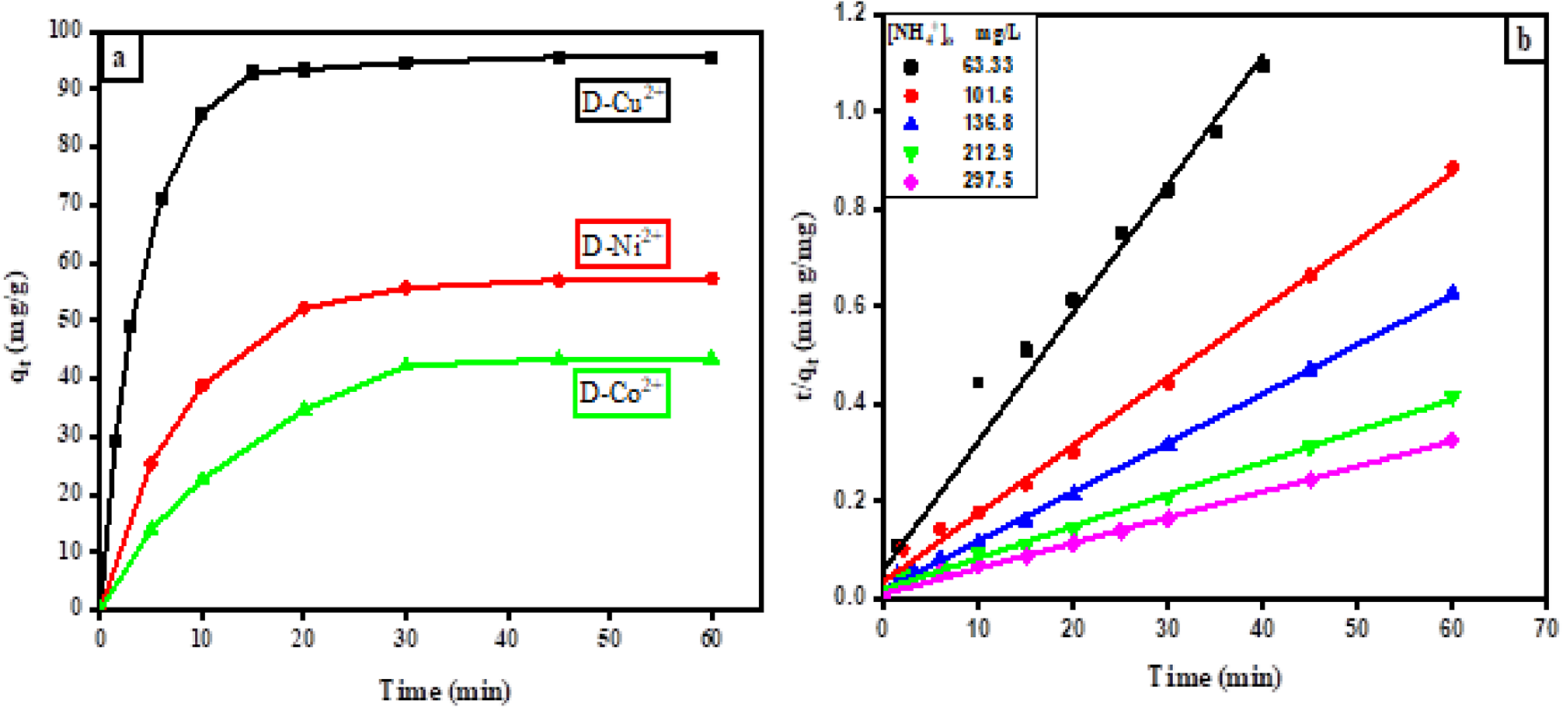
(**a**) The impact of contact time on the adsorption of NH_4_^+^, (adsorbent dose = 0.03 g, [NH_4_^+^]_o_ = 136.8 mg/L, 120 rpm and 30 °C). (**b**) Pseudo 2nd order kinetic plot of the NH_4_^+^ adsorption using (0.03 g) of D-Cu^+2^ at 30 °C.

The effectiveness of the removal of NH_4_^+^ from an aqueous solution using D-Cu^2+^, D-Ni^2+^, and D-Co^2+^ was compared utilizing an identical initial concentration of NH_4_^+^. It was noticed that the equilibrium adsorption capacity of D-Cu^2+^ (q_e_ = 95.58 mg/g) towards NH_4_^+^ was greater than that of D-Ni^2+^ (q_e_ = 57.29 mg/g) and D-Co^2+^ (q_e_ = 43.43 mg/g), as can be seen in (Fig. 3a). The highest performance of D-Cu^2+^ in removing NH_4_^+^ is due to the highest loaded amount of copper on D-H surface compared to nickel and cobalt^42,43^. The adsorption process of NH_4_^+^ on the loaded D-H was controlled by two techniques. One included the ion exchange between hydrogen ions of a sulfonated group attached to the polymeric resin and NH_4_^+^. The other represented the ligand exchange of NH_4_^+^ with hydrated water and formed a complex with transition metal cations loaded on the D-H surface. Since D-Cu^2+^ was represented as the best applicable adsorbent for removing NH_4_^+^ from aqueous solutions, detailed experiments aimed at studying its efficiency in getting rid of NH_4_^+^ were carried out under various experimental conditions.

The adsorption of NH_4_^+^ on D-Cu^2+^ is influenced by contact time, (Fig. 3a). Within the first 15 min, NH_4_^+^ was rapidly adsorbed onto D-Cu^2+^, reaching a plateau after 20 min. It is possible to attribute the rapid initial adsorption of NH_4_^+^ onto D-Cu^2+^ to a vast number of replaceable active sites, which promote accelerated ion exchange and complex formation rates. Then the adsorption process was continued at a slower rate until equilibrium was attained. This is due to the active sites on the surface became gradually occupied, and there was a noticeable decline in the rate of adsorption, which led to the equilibrium state^8^.

The maximum ammonia adsorption capacity (q_max_) of D-Cu^2+^ is comparable to that of other common NH_4_^+^ capture adsorbents, such as cation exchange resin, clay minerals, their modifications, nanocomposite, and Cu(II)-loaded adsorbents described in the literature, as seen in (Table 1). D-Cu^2+^ showed the highest NH_4_^+^ adsorption capacity within 20 min. This means that D-Cu^2+^ is a viable adsorbent for removing NH_4_^+^ from an aqueous solution.

**Table 1:** The maximum ammonia adsorption capacity of D-Cu^2+^ and several adsorbents are described in the literature.

Adsorbent	q_max_ (mg/g)	Conditions	References
Cation exchange resin	4.710	T = 303 K, t = 20 min	^30^
Clay mineral (vermiculite)	50.06	T = 298 K, pH = 7, t = 30 min	^66^
NaA zeolite (NZ)	23.27	T = 298 K, pH = 7, t = 6 h	^19^
NaCl modified vermiculite (Na–V)	7.079	T = 298 K, pH = 8, t = 8 h	^67^
Lithium titanate (LiT)	50.31	T = 298 K, pH = 7, t = 120 min	^68^
Magnetic nZVI/Z composite	19.88	T = 298 K, pH (3–10), t = 120 min	^22^
ZrO_2_/GO	84.47	T = 298 K, pH = 7, t = 30 min	^51^
Cu(II)-loaded D751 resin	5.390	T = 298 K, pH = 11, t = 90 min	^26^
CuHCF	34.92	T = 298 K, pH = 8, t = 1 h	^69^
CuHCF-WAC	47.07	T = 303 K, pH = 6.5, t = 24 h	^44^
D-Cu^2+^	280.9	T = 303 K, pH = 8.4, t = 20 min	This study

To numerically describe the adsorption kinetics and fit the experimental data of the adsorption, linear pseudo 1st model, linear pseudo 2nd order, and intraparticle diffusion models were evaluated according to (Supplementary Eqs. S1, S2, and S3), respectively. More information about these models was given in the online resource. The low value of the correlation coefficient of the pseudo-first-order model (R^2^ ≈ 0.95), as appeared in (Table 2), proved that this model is inappropriate and unsuitable for the prediction of the adsorption of NH_4_^+^ onto D-Cu^2+^. However, the pseudo 2nd order model has higher R^2^ values (> 0.99), which was nearly equal to one. Additionally, the calculated adsorption capacity (q_e,cal_) of the pseudo 2nd order model was extremely close to the experimental one (q_e_,_exp_). This indicates that the pseudo 2nd order model predicts the adsorption of NH_4_^+^ onto D-Cu^2+^ more accurately than the pseudo 1st order model, as presented in Fig. 3b. The pseudo 2nd order model's superior fit demonstrates how the adsorption mechanism is dependent on the ratio of adsorbent to adsorbate. According to the above results, the NH_4_^+^ adsorption by D-Cu^2+^ is a chemisorption process^44–48^.

**Table 2:** Parameters of kinetic models along with their correlation coefficient (R^2^) for the NH_4_^+^ adsorption onto D-Cu^2+^ (0.03 g) at 30 °C.

[NH_4_^+^]_0_ (mg/L)	Pseudo 1st order model					Pseudo 2nd order model				Intraparticle diffusion model					
	q_e(exp)_ (mg/g)	q_e(cal)_ (mg/g)	k_1_ (1/min)	R^2^	SSE	q_e(cal)_ (mg/g)	k_2_ (g/mg min)	R^2^	SSE	k_p1_ (mg/g min^1/2^)	R^2^	SSE	k_p2_ (mg/g min^1/2^)	R^2^	SSE
63.33	36.51	35.84 ± 0.21	0.113 ± 0.01	0.950	0.518	37.87 ± 0.01	0.011 ± 0.02	0.991	0.096	5.548 ± 1.11	0.961	4.682	2.981 ± 0.41	0.929	2.857
101.6	67.79	61.62 ± 0.29	0.178 ± 0.01	0.966	1.807	71.53 ± 0.04	0.006 ± 0.01	0.994	0.038	21.10 ± 0.31	0.999	0.144	0.915 ± 0.38	0.657	4.402
136.8	95.58	67.63 ± 0.21	0.163 ± 0.01	0.973	1.346	99.40 ± 0.01	0.005 ± 0.01	0.997	0.098	29.09 ± 2.71	0.983	3.139	0.738 ± 0.12	0.929	0.423
212.9	144.5	117.7 ± 0.20	0.141 ± 0.01	0.974	0.856	153.1 ± 0.02	0.003 ± 0.01	0.993	0.097	38.04 ± 1.98	0.995	2.593	1.893 ± 0.47	0.845	6.654
297.5	183.6	155.9 ± 0.12	0.146 ± 0.01	0.993	0.222	190.8 ± 0.01	0.002 ± 0.01	0.996	0.031	44.31 ± 1.33	0.998	1.357	2.963 ± 0.80	0.776	2.650

According to the intra-particle diffusion plot, which is depicted in (Supplementary Fig. S1), the adsorption mechanism is divided into two stages. The quick adsorption phase of the first phase exhibited a sharp slope. It was consistent with the liquid film's surface diffusion. The initial phase involved a straight line that failed to intersect with the point of origin. This shows that the adsorption of NH_4_^+^ occurred over the exterior surface. Furthermore, increasing the initial concentrations of NH_4_^+^ from 63.33 to 297.5 mg/L caused the adsorption rate to accelerate and increased values of kp_1_. Table 2 lists the values of kp_1_ and kp_2_, with kp_1_ being larger than kp_2_^45,49^.

### The effect of solution pH

**Figure 4:**
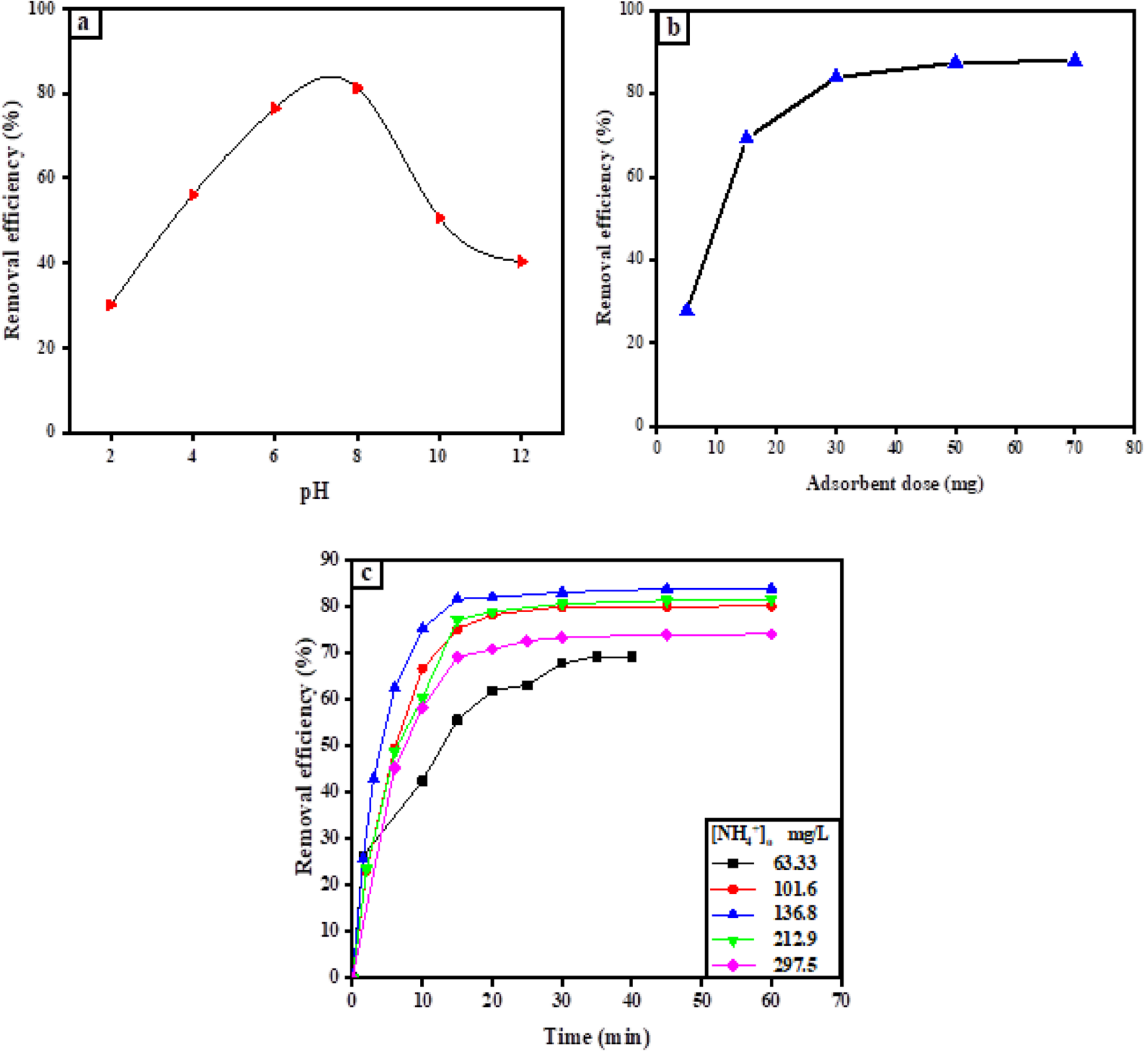
(**a**) The impact of initial pH on NH_4_^+^ removal ([NH_4_^+^]_o_ = 136.8 mg/L using 0.03 g of D-Cu^2+^, pH = 8.4 at 30 °C), (**b**) Effect of D-Cu^2+^ dose on the NH_4_^+^ removal efficiency ([NH_4_^+^]_o_ = 136.8 mg/L, pH = 8.4 at 30 °C), (**c**) The impact of the initial concentration of NH_4_^+^ on the removal efficiency with the contact time, (D-Cu^2+^ dose = 0.03 g, pH = 8.4 at 30 °C).

One of the most important factors in the removal of NH_4_^+^ by D-Cu^2+^ is the medium's pH. Because it plays an important role in the ratio of two forms of ammonia and the adsorbent surface. To evaluate the NH_4_^+^ removal percentage as a function of pH, the solution pH was changed from 2 to 12 using a universal buffer (Fig. 4a). However, the initial concentration of NH_4_^+^, the dose of D-Cu^2+^, and the temperature were kept constant. The pH_PZC_ of D-Cu^2+^ was determined to be around 5.5 in water, as shown in Supplementary Fig. S2. When the pH of the medium lower than 5.5 demonstrated low removal efficiency. This can be attributed to the surface was protonated and high concentration of H^+^ ions in the solution competed with the uptake of NH_4_^+^, lowering the adsorbed amount of NH_4_^+^ by D-Cu^2+^ through the ion exchange process^50^. The removal efficiency (R%) increased noticeably from 30.13 to 76.48% when the pH rose from 2 to 6. The highest removal efficiency was achieved at pH (6–8)^51,52^. Conversely, the R% of NH^4+^ dramatically dropped above pH 8.00, from 81.23% to 40.36% at pH 12. This is due to the Cu^2+^ on D-H surface becoming hydrolysis and producing Cu(OH)_2_, which decrease the uptake of NH^4+^ through ligand exchange and complex formation. The obtained results are completely consistent with our previous work^31^.

### Influence of D-Cu^2+^ dosage

The effect of D-Cu^2+^ dose on the removal efficiency of NH_4_^+^ is illustrated in (Fig. 4b). Variable doses of D-Cu^2+^ (5–70 mg) were used to investigate the effect of the adsorbent dosage. The removal efficiency of NH_4_^+^ significantly increased from 27.77 to 83.84% as the dose of D-Cu^2+^ was raised from 5 to 30 mg. The reason for this pattern is that, when the adsorbent dosage increased, a large number of exchangeable active adsorption sites became available. Furthermore, the rate of coordination complexation increased as the concentration of loaded copper on the D-H surface increased^25^. The occupation of extra-active sites caused the removal efficiency to reach the plateau at 30 mg of D-Cu^2+^ and did not significantly increase up to 70 mg, these results agree with that obtained by Mousavi et al.^51^. As a result, a 30 mg dose of D-Cu^2+^ was chosen to study the influence of the other experimental factors on the uptake of NH_4_^+^ by D-Cu^2+^.

### Effect of initial concentration of ammonia

The effect of the initial concentration of NH_4_^+^ was investigated by varying its concentration from 63.33 to 297.5 mg/L, (Fig. 4c). When the NH_4_^+^ concentration raised from 63.33 to 136.8 mg/L, the NH_4_^+^ removal rate utilizing D-Cu^2+^ (0.03 g) during the stated contact time (20 min) got up to 83.84%. This is caused by an increase in the NH_4_^+^ concentration gradient in the solution, which creates a strong driving force for mass transfer^53^. Moreover, the saturation of the active adsorption sites (Cu(II) ions) with the adsorbed NH_4_^+^ to form Cu(II)-ammine complexes, and then the system reached an equilibrium state^51^. When [NH_4_^+^]_o_ increased beyond 136.8 mg/L, the removal of NH_4_^+^ decreased. This occurred due to the lack of free adsorption sites on the D-Cu^2+^, and all Cu^2+^ ions were bound to NH_4_^+^ molecules.

### Adsorption isotherms

Four isothermal models were applied to analyze the equilibrium data for NH_4_^+^ adsorbed on D-Cu^2+^, involving Langmuir, Freundlich, Temkin, and D-R. The linear and non-linear plots of the Freundlich and Langmuir isotherms were presented in Supplementary Figs. S3, S4, S5, and S6, respectively. All parameters derived from the isotherm models were reported in Table 3, together with the values of (R^2^). A glance at Table 3, revealed that the non-linear Langmuir model was the best model to describe the adsorption of NH_4_^+^ on D-Cu^2+^, particularly at higher temperatures. A dimensionless constant known as the separation factor (R_L_), which describes the Langmuir isotherm, is written as R_L_ = 1/(1 + K_L_C_e_)^54^. According to the information in (Table 3), the values of (R_L_) in the range of 0.12–0.35 showed favorable Langmuir adsorption, at all temperatures. The values of (q_max_) decreased from 333.6 to 280.9 mg/g as the temperature rose from 293 to 303 K, which was consistent with an exothermic reaction^55^.

**Table 3:** Isotherm parameters and the correlation coefficient, R^2^ for the adsorption of NH_4_^+^ onto (0.03 g) D-Cu^2+^.

T (K)	Langmuir isotherm					Freundlich isotherm				Temkin isotherm				Dubinin-Radushkevich isotherm				
	q_max_ (mg/g)	K_L_ (L/mg)	R_L_	R^2^	SSE	K_F_ (mg/g)	1/n	R^2^	SSE	K_T_ (L/mg)	B_1_ (J/mol)	R^2^	SSE	q_m_ (mg/g)	B/10^–4^ (mol^2^ kJ^-2^)	*E* (kJ/mol)	R^2^	SSE
Linear form																		
293	72.67 ± 0.01	0.024 ± 0.20	0.123	0.445	0.026	0.125 ± 1.82	0.472 ± 0.59	0.811	0.215	0.133 ± 5.79	95.59 ± 1.72	0.912	8.501	219.9 ± 0.08	-0.656	87.32 ± 0.01	0.964	0.023
303	163.1 ± 0.01	0.013 ± 0.22	0.205	0.162	0.053	0.634 ± 1.96	0.659 ± 0.61	0.674	0.376	0.140 ± 3.96	79.66 ± 1.12	0.944	4.500	195.6 ± 0.07	-0.662	86.90 ± 0.01	0.964	0.021
313	94.16 ± 0.01	0.010 ± 0.20	0.252	0.638	0.042	0.122 ± 1.01	0.582 ± 0.27	0.930	0.086	0.054 ± 6.03	100.5 ± 1.51	0.936	4.614	189.2 ± 0.13	-2.224	47.42 ± 0.01	0.919	0.058
Non-linear form																		
293	333.6 ± 1.37	0.023 ± 0.01	0.128	0.915	11.22	14.98 ± 0.80	1.595 ± 0.38	0.855	13.98	–	–	–		–	–	–	–	
303	280.9 ± 0.68	0.025 ± 0.01	0.119	0.989	5.671	16.34 ± 0.68	1.772 ± 0.35	0.897	8.156	–	–	–		–	–	–	–	
313	421.3 ± 0.52	0.006 ± 0.01	0.359	0.944	7.039	3.823 ± 0.27	1.213 ± 0.25	0.893	7.756	–	–	–		–	–	–	–	

Both Temkin and D-R models predicted that the chemisorption behavior predominated for NH_4_^+^ adsorption. It was found that the value of mean sorption energy (E), which was computed from the data of the D-R model, was more than 40 kJ/mol, suggesting that chemical action is the predominant adsorption mechanism^21^. This proves that removing NH_4_^+^ from an aqueous solution using D-Cu^2+^ included coordination complexation that formed between aqueous ammonia and the loaded Cu(II) ions on the surface of D-H.

### Adsorption thermodynamics

At three distinct temperatures (293, 303, and 313 K), the adsorption of NH_4_^+^ on D-Cu^2+^ was investigated. The enthalpy change ($\Delta {\text{H}}_{\text{ads}}$) and entropy change ($\Delta {\text{S}}_{\text{ads}}$) of adsorption were calculated using the Van’t Hoff equation (Supplementary Eq. S4). However, the change in Gibbs-free energy of adsorption (ΔG_ads_) was calculated from equations (Supplementary Eqs. S5, S6). Supplementary Figure S7 and Table 4, respectively, provided the Van’t Hoff plot and the values of thermodynamic parameters. As seen in Table 4, the value of K_d_ decreased as the temperature increased. This demonstrates the exothermic characteristics of the adsorption process^27^. At all reaction temperatures, ΔG_ads_ showed negative values for the removal of NH_4_^+^ using D-Cu^2+^, confirming the spontaneous and favorable nature of the adsorption process^8^. The value of ΔH_ads_ was also negative, indicating exothermic adsorption process. Additionally, the negative value of ΔS_ads_, reveals the randomness at the solid/liquid interface and entropy decreased throughout the NH_4_^+^ adsorption process^56^.

**Table 4:** Thermodynamic parameters of NH_4_^+^ adsorbed onto (0.03 g) D-Cu^2+^.

Temp (K)	K_d_ (L/g)	ΔH_ads_ (kJ/mol)	ΔS_ads_ (J/mol K)	ΔG_ads_ (kJ/mol)
293	4.804			− 4.072
303	4.322	− 30.31	− 89.55	− 3.176
313	2.153			− 2.281

### Effect of coexisting ions

**Figure 5:**
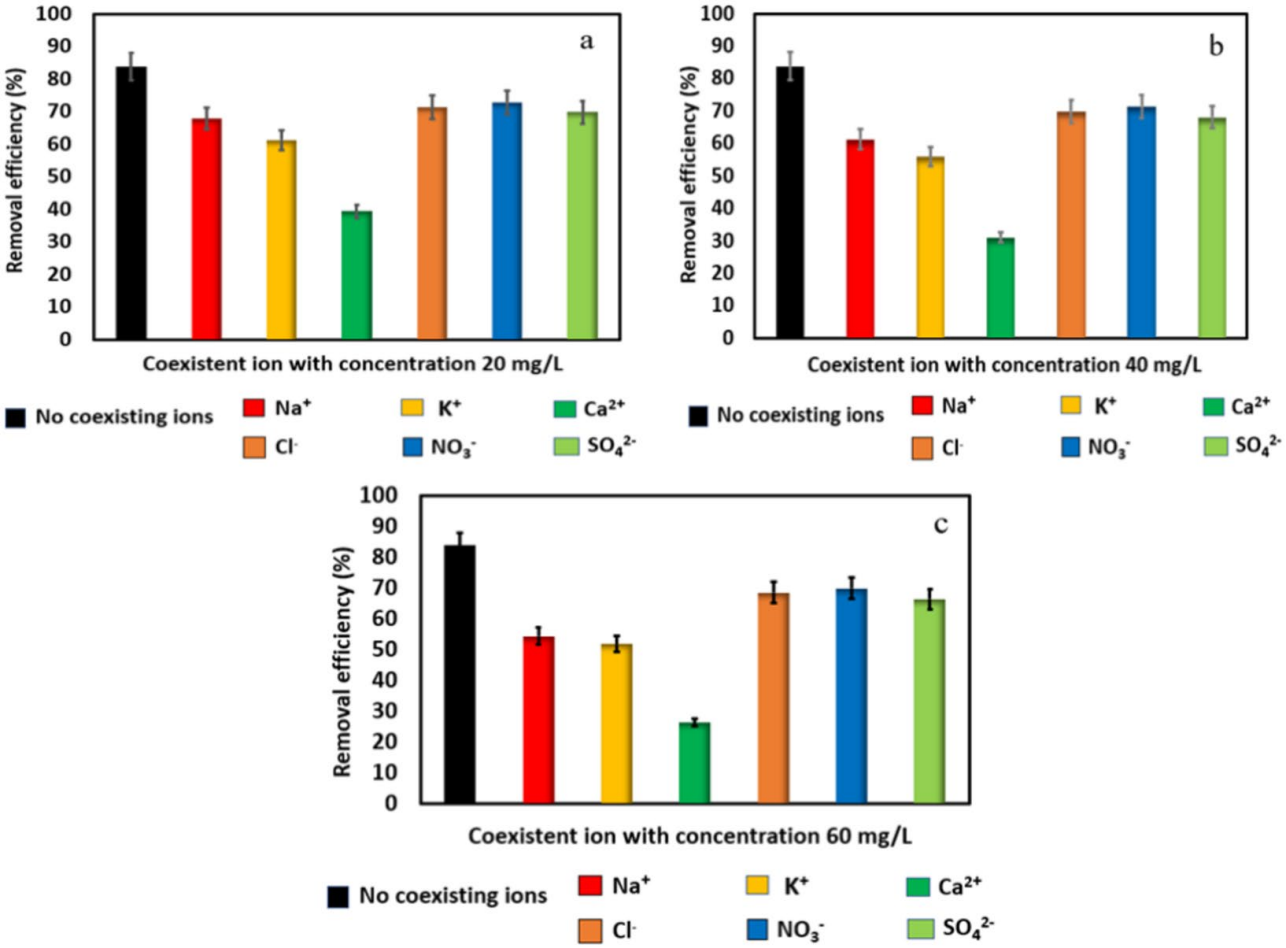
(**a**–**c**) Effect of coexisting ions with different concentrations on NH_4_^+^ adsorption, [NH_4_^+^]_o_ = 136.8 mg/L using D-Cu^2+^ (0.03 g) at 30 °C.

Industrial and agriculture wastewater typically contains inorganic salts; these ions may compete with ammonia for adsorption sites on the D-Cu^+2^ surface. To assess the impact of these species (cations and anions) on the adsorption process, several ions, including Na^+^, K^+^, Ca^2+^, Cl^−^, NO_3_^−^, and SO_4_^2−^ were spiked. Experimental batches were carried out using 0.03 g of D-Cu^2+^ and 136.8 mg/L of NH_4_^+^. After 60 min. of adsorption time, the residual NH_4_^+^ concentration was then measured. In the presence of Na^+^, K^+^, and Ca^2+^, the R % of NH_4_^+^ decreased from 83.84 to 67.84%, 61.30%, and 39.43%, respectively. This was mostly due to the competitive adsorption between these cations with NH_4_^+^ on D-Cu^2+^ during the ion exchange process in simulated wastewater. Ca^2+^ ion has a high valence form, causing its influence to be notably stronger than that of other cations^57,58^. As can be seen in Fig. 5a–c, increasing the concentration of cations increases the competition between these cations and NH_4_^+^, which leads to a gradual decrease in the amount of NH_4_^+^ uptake^59^. In the presence of different anions, such as Cl^−^, NO_3_^−^, and SO_4_^2−^, the R % of NH_4_^+^ decreased to 71.30%, 72.91%, and 69.84%, respectively. Therefore, these anions had a little minor impact on the removal efficiency of NH_4_^+^. According to these results, which are shown in Fig. 5a–c, both cations and anions have a limited influence except the Ca^2+^ ion on the removal efficiency of NH_4_^+^ by D-Cu^2+^. Since the majority of NH_4_^+^ removal occurs as a result of coordination complexation with copper ions at the D-H surface.

### Removal of ammonia from synthetic wastewater

A synthetic wastewater sample that was prepared according to the procedures in the experimental part was used to evaluate the performance of D-Cu^2+^ for the removal of NH_4_^+^. Due to interference from other ions present in the industrial wastewater, (0.03 g) of D-Cu^2+^ only succeeded in removing 44.35% of NH_4_^+^ from the effluent after 60 min, this is lower than its removal efficiency in the aqueous medium (83.83%).

### Application of the catalytic performance of D-Cu(II)-ammine composite for degradation of dyes

**Figure 6:**
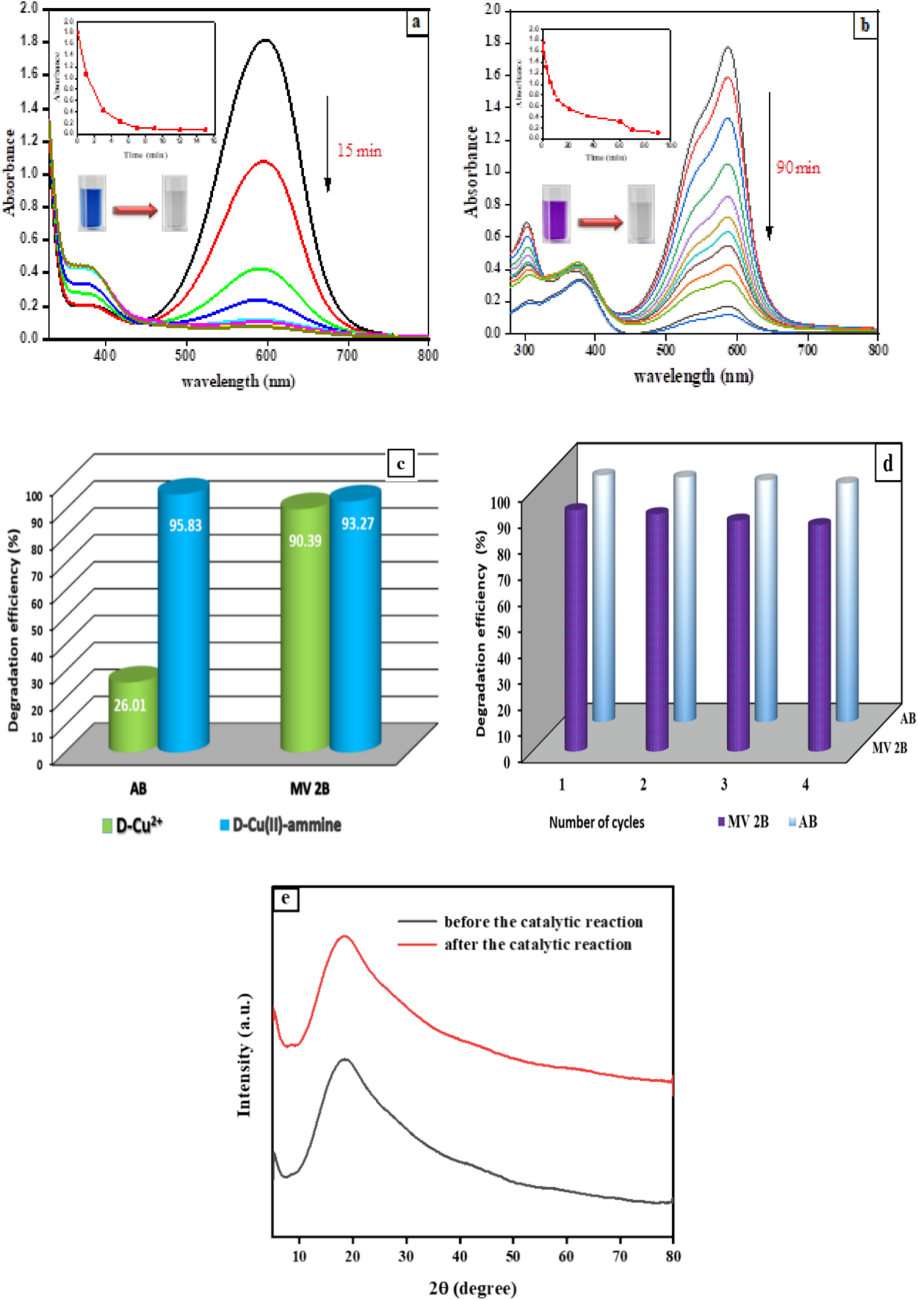
UV-vis spectra, as a function of time during catalytic decomposition of dyes in aqueous solution using 0.05 g of D-Cu(II)-ammine composite, [H_2_O_2_]_o_ = 0.01 mol/L at 30 °C. (**a**) [AB]_o_ = 7.5 × 10^–5^ mol/L, (**b**) [MV]_o_ = 1.86 × 10^–4^ mol/L, (**c**) The degradation efficiency of the AB and MV2B by D-Cu^2+^ (0.05 g) and D-Cu(II)-ammine composite (0.05 g), [H_2_O_2_]_o_ = 0.01 mol/L at 30 °C, and (**d**) D-Cu(II)-ammine composite recycling during the degradation of AB and MV2B with H_2_O_2_. (**e**) XRD of D-Cu(II)-ammine composite before and after the consecutive four catalytic cycles.

Dyes have been globally produced in huge quantities as a result of substantial growth in modern industries for use in dying fabrics, leather, paper, plastics, cosmetics, printing, food, pharmaceutical industry, and others^60^. However, they impact the survival of organisms. Large amounts of effluent from the dyeing process color the wastewater. This effluent impairs photosynthesis and negatively affects human and marine organisms' health^61^. Optimization of the D-Cu(II) ammine composite obtained from the NH_4_^+^ adsorption process using D-Cu^2+^ was performed. This product was used as a catalyst to break down two types of dyes (AB and MV2B) present in polluted water. The decomposition of the two dyes occurred in the presence of an environmentally friendly oxidizing agent, H_2_O_2_. The UV-vis spectra and time-dependent absorbance decreases of the two dyes are shown in Fig. 6a,b. The oxidative degradation of AB and MV2B in the presence of H_2_O_2_ using D-Cu^2+^ and D-Cu(II)-ammine individually as catalysts was illustrated in Fig. 6c. Within 15 and 90 min, it was found that approximately 95.83% and 93.27% of AB and MV2B were degraded, respectively in the presence of D-Cu(II)-ammine composite. However, only 26.01% and 90.39% of AB and MV2B were degraded, respectively in the presence of D-Cu(II) as a catalyst. These results showed that the D-Cu(II)-ammine composite had better catalytic activity than D-Cu^2+^ for the degradation of both AB and MV2B. Moreover, CO_2_ evolved from the reaction of AB and MV2B with H_2_O_2_ in the presence of D-Cu(II)-ammine composite was captured by an aqueous solution of barium hydroxide. As the reaction was carried out, a white precipitate of BaCO_3_ emerged, signifying the generation of CO_2_ as a catalytic decomposition product of AB and MV2B.

### Recycling of D-Cu(II)-ammine composite

From a practical standpoint, lowering the processing cost is critical for ensuring sustainable economic growth. As a result, the recovery and reusability of the D-Cu(II)-ammine composite as a catalyst were assessed utilizing the oxidative degradation of AB and MV2B with H_2_O_2_ as an experimental reaction. After the reaction ended, the catalyst was thoroughly rinsed with distilled water and H_2_O_2_ solution, dried, and then reused in the subsequent reaction cycle. To demonstrate the excellent catalytic activity and stability of the catalyst, four successive cycles of catalyst reusability were run. Figure 6d indicated a very slight loss in catalytic activity within the four cycles. From the first to the fourth cycle, the degradation efficiency of the AB and MV2B varied from 94.64 to 91.57% and 92.62 to 86.92%, respectively. This finding demonstrates that the D-Cu(II)-ammine composite is stable and may be recycled effectively several times with only a slight decrease in its catalytic activity up to four cycles. Moreover, XRD pattern (Fig. 6e) of D-Cu(II)-ammine composite before and after four reusability cycles indicates no change in its structure. This confirms the stability of the D-Cu(II)-ammine composite under the reaction conditions and can be a good candidate for other catalytic applications.

## Experimental

### Materials and chemicals

Ion exchange resin, Dowex-50WX8 (D-H) was obtained from (DOW Chemical Co., USA). The physical and chemical properties of D-H are listed in Table 5.

**Table 5:** Specifications of Dowex-50X8 (D-H).

Specification	Dowex-50WX8 (D-H)
Appearance (color)	Faint yellow
Type	Strongly acidic cation exchange resin
Functional group	(–SO_3_–H) sulfonic acid as a surface active group
Matrix structure	Cross-linked styrene-divinyl benzene (ST-DVB) copolymer
Ionic form	(H^+^) form
Shape	granules
Particle size	100–200 dry mesh
Moisture content	50–58%
Density	0.8 g/mL
Operating pH	0–14
Thermal stability	up to 100 °C
Total exchange capacity	> 1.7 meq/mL (wet)
Cross-linkage	8% divinyl benzene (DVB)

Sodium nitroprusside dihydrate and Thymol were purchased from (LANXESS AG, Germany) and used as received. Sodium hypochlorite (4–5%), ammonium hydroxide (36%), acetic acid (99%), sodium chloride, potassium chloride, calcium chloride, potassium nitrate, and potassium sulfate were purchased from (ADWIC, Egypt). Hydrochloric acid (30%) and phosphoric acid (99%) were purchased from (SDFCL, India). Sodium hydroxide, sodium carbonate, sodium hydrogen carbonate, boric acid, cobalt(II) chloride, nickel(II) chloride, copper(II) sulfate, and barium hydroxide were obtained from (Sigma-Aldrich, Egypt). Hydrogen peroxide (50%) was obtained from (Merck, Germany). Aniline blue (AB) and Methyl violet 2B (MV 2B) were obtained from (Sigma-Aldrich, Egypt) and used as received, Table S1 contains information on these dyes. Distilled water was used for preparing the standard solutions.

### Instrumentation

The structure and morphology of Cu(II) supported on D-H resin have been evaluated using several techniques. FT-IR analysis was performed using (JASCO FT-IR-4100, Japan) with a resolution of 2 cm^−1^ using potassium bromide within the wavenumber range of 4000–400 cm^−1^. Thermogravimetric analysis (TGA) was performed under N_2_ at a scanning rate of 10 °C min^−1^ using a thermal analyzer (SDT Q600 V20.9 Build 20, USA). X-ray powder diffraction (XRD) analysis was obtained by (GNR APD 2000 PRO, Germany). Nickel-filtered Cu Kα radiation beam with wavelength (λ = 1.540 Å), radiation operated at 40 kV and 30 mA at scanning rate of 0.03°/min over a continuous range 2θ of 5°–80°. Scanning electron microscope (SEM) measurements were performed with (JEOL and JSM-6510LV, Japan). The energy dispersive X-ray spectroscopy (EDX) analysis was investigated using an X-Max SDD Detector attached to an SEM device that delivers high-quality EDX data (Oxford X-Max 20, USA). A high-performance double-beam spectrophotometer with an electronic temperature controller (SPECORD 210 PLUS, Analytic Jena, Germany) was used to perform the UV-vis measurements. The concentration of metals was determined by an inductively coupled plasma optical emission spectrometer (ICP-OES) Optima 7000 DV with a double monochromator and a simultaneous CCD array detector (Perkin Elmer, USA). The pH of the medium was adjusted using a pH bench meter (AD1030, Adwa, Hungary). A water shaker thermostat (Julabo D-7633 Seelbach, Germany) was used to shake the mixtures during the adsorption process.

## Methods

### Activation of Dowex-50WX8 resin

To eliminate any fine particles and unwanted substances, A suitable quantity of D-H was thoroughly washed with distilled water. Afterward, it was filtered and dried in air before use. To enhance the exchanger's capacity, it was treated with 0.1 M HCl, then rinsed with distilled water to eliminate any excess chloride ions that were ascertained by the AgNO_3_ test, and then dried at room temperature overnight^62^.

### Preparation of metal ions supported on Dowex-50WX8 resin (D-M^n+^)

500 mL of a copper(II) sulfate solution with concentration (0.5 M) were utilized to disperse 5 g of D-H. To reach the equilibrium condition, the mixture was magnetically agitated for 24 h. After that, it was extensively filtered and rinsed with distilled water repeatedly until no Cu(II) ions were found in the filtrate, and this was confirmed by ICP-OES measurements. The loaded amount of Cu(II) ions onto the resin was determined by measuring the concentration of copper ions before and after the loading process using an ICP-OES spectrometer. The D-Cu^2+^ product was dried in an oven at 50 °C for 12 h. Under the same conditions, the same method was used to prepare D-Ni^2+^ and D-Co^2+^^31^.

### Ammonia adsorption experiments

The removal of NH_4_^+^ from an aqueous solution was studied using cationic ligand exchange material namely, Dowex-50WX8 (D-M^2+^). 0.03g of D-M^2+^ was mixed with 25 mL of a solution of NH_4_^+^, its initial concentration 136.8 mg/L in several stoppered Erlenmeyer flasks. All of these vessels were quickly agitated at 120 rpm for a specific time in a shaking water bath with a controlled thermostat at 30 °C.

The removal efficiency R (%), and the adsorption capacity of NH_4_^+^ at equilibrium q_e_ (mg/g) were calculated using Eqs. (2) and (3), respectively.

2 $$\text{R}\left({\%}\right)= \frac{{\text{C}}_{\text{o}}-{\text{C}}_{\text{e}}}{{\text{C}}_{\text{o}}}\text{ x }100$$

3 $${\text{q}}_{\text{t}}= \frac{{(\text{C}}_{\text{o}}-{\text{C}}_{\text{e}})\text{ x V}}{\text{m}}$$

where C_o_ and C_e_ (mg/L) represent the initial and equilibrium NH_4_^+^ concentrations, respectively, V (L) is the volume of solution, and m (g) is the mass of D-M^2+^ that was used. The indothymol blue method (ITB) was used to determine the NH_4_^+^ concentration^63^. The absorbance of the formed blue color was measured at λ_max_ = 693 nm using a UV-vis spectrometer (SPECORD 200 PLUS). For D-Cu^2+^ as an adsorbent, several experimental parameters were examined to optimize their impact on the adsorption efficiency. Such as the effect of pH (2–12) using a universal buffer, temperature (293–313 K), contact time (2–60 min), the initial concentration [NH_4_^+^]_o,_ and the effect of coexisting ions.

### Adsorption kinetics and isotherms

The kinetic parameters were evaluated through the linear forms of three kinetic models namely, linear pseudo 1st order, linear pseudo 2nd order, and intraparticle diffusion models Supplementary Eqs. S1, S2 and S3, respectively^64^. Moreover, the equilibrium adsorption data was checked using linear and non-linear forms of Freundlich (Supplementary Eqs. S7 and S8, respectively) and Langmuir models (Supplementary Eqs. S9 and S10, respectively)^65^. Additionally, the experimental data were introduced in Temkin and Dubinin-Radushkevich (D-R) isotherms (Supplementary Eqs. S11 and S12, respectively).

### Removal of ammonia from prepared synthetic wastewater

5 g of commercial urea fertilizer was mixed in 100 mL of distilled water to create synthetic wastewater. This solution additionally contained a specific amount (0.01 g) of ammonium chloride, calcium chloride, potassium chloride, and sodium chloride. 0.03 g of D-Cu^2+^ was added into 25 mL of synthetic wastewater and then shaken at 120 rpm and 30 °C using a thermostatic shaking water. After 60 min, the residual NH_4_^+^ concentration was determined by using indothymol blue spectrophotometric method.

### Reusability of D-Cu(II)-ammine composite as a catalyst for dyes degradation

The recovery and reusability of D-Cu(II)-ammine composite after washing with distilled water several times and drying in an oven at 50 °C for 12 h were studied. This composite was used for the catalytic degradation of AB and MV2B in the presence of H_2_O_2_. The optimum reaction mixture was set up in a 100 mL Erlenmeyer flask. In these flasks, 0.05 g of the dried D-Cu(II)-ammine composite was added to a certain volume of AB (1 × 10^–3^ mol/L) and/or MV2B (2.4 × 10^–3^ mol/L) dyes and 1 mL of H_2_O_2_ with concentration of 0.2 mol/L. The flasks were put in a water shaker thermostat at 30 °C ± 0.2 °C and agitated at 120 rpm for a given period. The decrease in absorbance of each non-reacted dye solution was measured using a UV-vis spectrometer (SPECORD 200 PLUS) at λ_max_ = 598 nm and λ_max_ = 588 nm, respectively. The measurements were performed continuously until there was no further decrease in the absorbance.

### Consent to participate

All the authors agreed to participate in this work.

## Conclusion

To improve the efficiency and selective recovery of NH_4_^+^ from wastewater, cationic exchange resin, Dowex-50WX8 (D-H) was loaded with three transitional metal cations. 1 g of D-H was loaded with 296 mg/g, 90 mg/g, and 60 mg/g of Cu(II), Ni(II), and Co(II), respectively. The prepared Cu(II)-loaded D-H was characterized by various techniques. The ammonia removal percentage proceeded in the following order: D-Cu^2+^  > D-Ni^2+^  > D-Co^2+^. The adsorption of ammonia onto D-Cu^2+^ was consistent with the pseudo 2nd order model and predominantly controlled by the chemisorption process. The best isotherm model was the non-linear Langmuir, which yielded the highest equilibrium adsorption capacity (q_max_ = 280.9 mg/g) at pH = 8.4, and 303 K within 20 min. The adsorption process was exothermic, decreasing in entropy, and spontaneous reaction. The predominant mechanism for demonstrating the NH_4_^+^ adsorption process is the chemical behavior through coordinative complexation. The D-Cu(II)-ammine composite resulting from the NH_4_^+^ adsorption onto D-Cu^2+^, was used as a catalyst for the degradation of AB and MV2B with H_2_O_2_ as an oxidant. The D-Cu(II)-ammine composite is stable and may be recycled effectively several times with only a slight decrease in its catalytic activity up to four cycles.

### Supplementary Information

Supplementary Information.

## Data Availability

All the data and materials are available in the manuscript.
