# Supplementary material for: Copper supported Dowex50WX8 resin utilized for the elimination of ammonia and its sustainable application for the degradation of dyes in wastewater
Source: Sci Rep. 2024 Aug 27;14:19884. doi: 10.1038/s41598-024-69839-w (PMC11349949; doi:10.1038/s41598-024-69839-w)
Supplement: Supplementary file 1 — Supplementary Information. [file 41598_2024_69839_MOESM1_ESM.docx]

**Copper supported Dowex50WX8‎ resin utilized for the elimination of ammonia and its sustainable application for the degradation of dyes in wastewater**

*Mohamed M. Khamis*, Abeer S. Elsherbiny*, Ibrahim A. Salem****,*** *Marwa A. El-Ghobashy*

*Chemistry Department, Faculty of Science, Tanta University, Tanta 31527, Egypt*

*Corresponding Authors at Chemistry Department, Faculty of Science, Tanta University, Tanta 31527,

E-mail address: [abeer.elsherbiny@science.tanta.edu.eg](mailto:abeer.elsherbiny@science.tanta.edu.eg), [abeer.elsherbiny@yahoo.de](mailto:abeer.elsherbiny@yahoo.de) (A. S. Elsherbiny).

E-mail address: [mohamed.khamis@science.tanta.edu.eg](mailto:mohamed.khamis@science.tanta.edu.eg) (M. M. Khamis).

**Kinetics models**

The kinetic parameters were evaluated through the linear forms of three kinetic models namely, pseudo 1^st^ ‎order, pseudo 2^nd^ ‎order, and intraparticle diffusion models Eqs. (S1), (S2) and (S3), respectively ^1^.

$\ln\left( q_{e}-q_{t} \right)=lnq_{e}-k_{1} \left( 1 \right)$

$$\frac{t}{q_{t}}=\frac{1}{k_{2}q_{e}^{2}}+\frac{t}{q_{e}} \left( 2 \right)$$

$q_{t}=k_{p}t^{1/2}+C (3)$

Where, q_e_ and q_t_ (mg/g) represent the quantity of NH_4_^+^ adsorbed at equilibrium process and time t, respectively. The rate constants for pseudo 1^st^ ‎order, pseudo 2^nd^ ‎order, and intraparticle diffusion are k_1_ (1/min), k_2_ (g/mg min), and k_p_ (mg/g min^1/2^), respectively. C (mg/g) is a constant term that represents the thickness of the boundary layer.


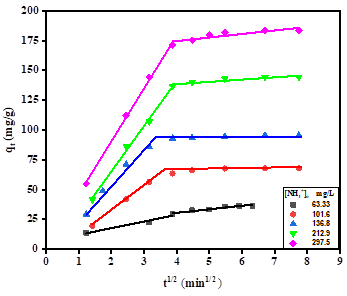


**Figure S1.** The plot of Intra-particle diffusion for the NH_4_^+^ adsorption onto (0.03 g) of D-Cu^+2^ at 30 °C.

**Determination of the point-zero charge (pH_PZC_)**

Using the pH drift method, the pH of the prepared D-Cu^2+^ at the point of zero charge (pH_PZC_) was established ^2^. Briefly, 0.1 M NaCl solutions with pH values varying from 2 to 12, were adjusted with (0.1 M HCl and NaOH solutions). Then, 0.05 g of the as-prepared D-Cu^2+^ was added to each 25 mL of 0.1 M NaCl solution of a pH-adjusted. The solutions were shaken in stoppered Erlenmeyer flasks at room temperature for 24 h. The final pH values of the supernatant liquid were measured. By plotting the initial pH vs. the final pH, the point at which the straight line of (pH_initial_ = pH_final_) intersects this curve is estimated to be pH_PZC_, as shown in (Fig S2) ^3^. The pH_PZC_ of D-Cu^2+^ was determined to be around 5.5.


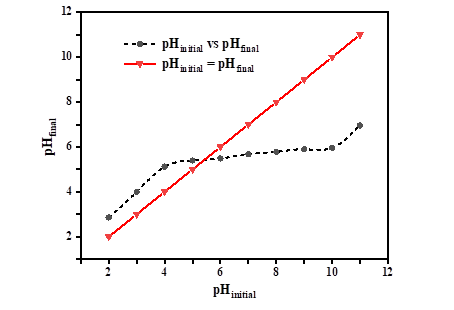


**Figure S2.** Zero-point charge of D-Cu^2+^.


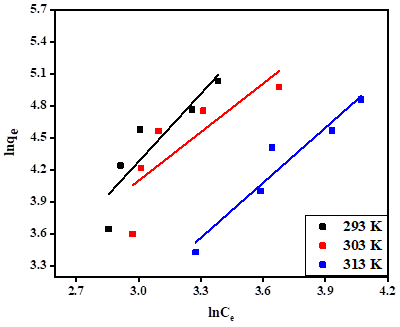


**Figure S3.** A linearized plot of Freundlich isotherm.

**
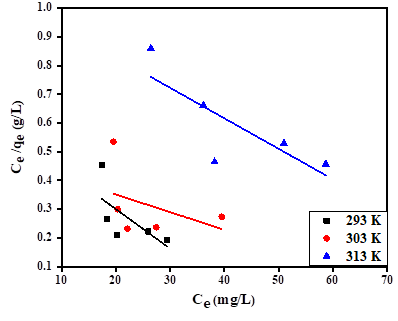
**

**Figure S4.** A linearized plot of Langmuir isotherm.


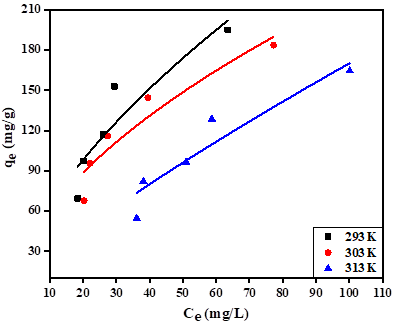


**Figure S5.** Non-linearized plot of Freundlich isotherm.

**
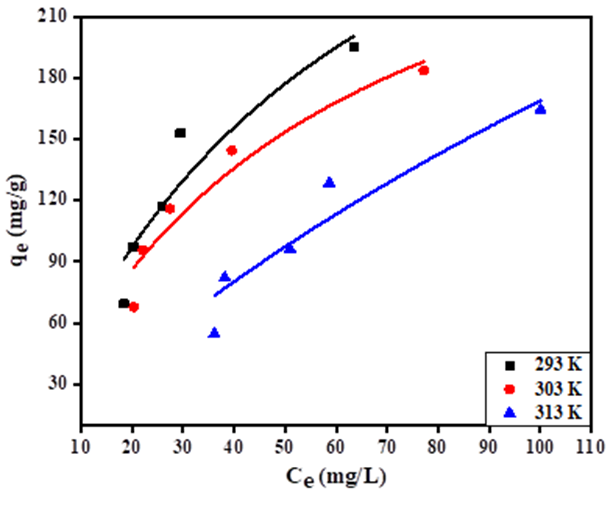
**

**Figure S6.** Non-linearized plot of Langmuir isotherm.

**Thermodynamics parameters**

The parameters of adsorption thermodynamics can be calculated by introducing the experimental data at three different temperatures into the following equations ^4^.

$$\ln K_{d}= -\frac{\Delta H_{\mathrm{ads}}}{\mathrm{RT}}+\frac{{\Delta S}_{\mathrm{ads}}}{R} (4)$$

$$\Delta G_{\mathrm{ads}}= -RT\ln K_{d} \left( 5 \right)$$

$$\Delta G_{\mathrm{ads}}=\Delta H_{\mathrm{ads}}-T\Delta S_{\mathrm{ads}} \left( 6 \right)$$

Where; K_d_ is the distribution coefficient (K_d_ = q_e_/C_e_), ΔG_ads_ is the change in Gibbs-free energy of the adsorption process, (kJ/mol) is the enthalpy change, and (J/mol K) is the entropy change.


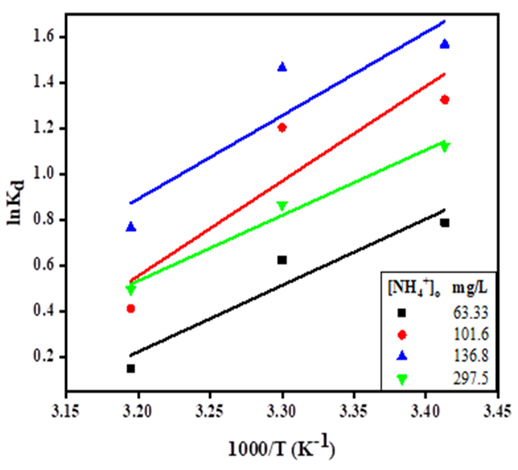


**Figure S7.** Van’t Hoff plot for the NH_4_^+^ adsorption using (0.03 g) of D-Cu^+2^.

| **Dye** | **Type** | **λ_max_ (nm)** | **Chemical Formula** | **MW** | **Structure** |
| --- | --- | --- | --- | --- | --- |
| Aniline blue (AB) | anionic | 595 | C_32_H_25_N_3_Na_2_O_9_S_3_ | 737.72 |  |
| Methyl violet 2B (MV 2B) | cationic | 582 | C_24_H_28_ClN_3_ | 393.96 |  |

**Table S1.** Information of dyes under investigation.

**Isotherm models**

The equilibrium adsorption data was checked using linear and non-linear forms of Freundlich (Eqs. (S7) and (S8), respectively) and Langmuir models (Eqs. (S9) and (S10), respectively). Additionally, the experimental data were introduced in Temkin and Dubinin-Radushkevich (D-R) isotherms (Eqs. (S11) and (S12), respectively).

$$\ln q_{e}= \frac{1}{n}\ln C_{e}+\ln K_{F} (7)$$

$$q_{e}=K_{F}C_{e}^{\frac{1}{n}} (8)$$

$$\frac{C_{e}}{q_{e}}=\frac{C_{e}}{q_{\max}} + \frac{1}{q_{\max}K_{L}} (9)$$

$$q_{e}= q_{\max} \left( \frac{K_{L}C_{e}}{1+ K_{L}C_{e}} \right) (10)$$

$q_{e}= B_{1}\mathrm{Ln}K_{T}+B_{1}\mathrm{Ln}C_{e} \left( 11 \right)$

$$\mathrm{Ln}q_{e}= \mathrm{Lnq}_{m}-B\varepsilon^{2} (12)$$

Where, C_e_ (mg/L) is the concentration of NH_4_^+^ at equilibrium, q_max_ (mg/g) is the maximum adsorption capacity of NH_4_^+^, and K_L_ (L/mg) is Langmuir adsorption constant that is related to the adsorption energy ^5^. K_F_ (mg/g) and 1/n are Freundlich constants related to the adsorption capacity and adsorption intensity, respectively. K_T_ (L/mg) is Temkin equilibrium constant connected to the maximum binding energy, and B_1_ (J/mol) is a constant representing the heat of adsorption which is calculated from the following ‎expression B = RT/b; R is the gas constant, T is the absolute temperature (K), and b is the adsorption potential ^6^. q_m_ is the monolayer capacity (mg/g), and ε is the Polanyi’s potential. The value of ε can be written as: ε = RT ln[1 + 1/C_e_]

The value of B gained from the slope of D-R’s plot is utilized to calculate the mean sorption energy (E, kJ/mol) which was obtained from the following equation:

**References**

1 Wu, X. *et al.* Adsorption characteristics and mechanism of ammonia nitrogen and phosphate from biogas slurry by Ca^2+^-modified soybean straw biochar. *PLOS ONE* **18**, e0290714, doi:<https://doi.org/10.1371/journal.pone.0290714> (2023).

2 Sayed, N. S. M., Ahmed, A. S. A., Abdallah, M. H. & Gouda, G. A. ZnO@ activated carbon derived from wood sawdust as adsorbent for removal of methyl red and methyl orange from aqueous solutions. *Sci. Rep.* **14**, 5384, doi:10.1038/s41598-024-55158-7 (2024).

3 Tahir, H., Anwer, M., Khan, S. & Saad, M. Enhancement of adsorption and photocatalytic activity of MgO nanoparticles for the treatment of textile dye using ultrasound assisted process by Response Surface Methodology. *Desalination and Water Treat.* **319**, 100429, doi:<https://doi.org/10.1016/j.dwt.2024.100429> (2024).

4 Elsherbiny, A. S. Adsorption kinetics and mechanism of acid dye onto montmorillonite from aqueous solutions: Stopped-flow measurements. *Appl. Clay Sci.* **83-84**, 56-62, doi:10.1016/j.clay.2013.07.014 (2013).

5 Doekhi-Bennani, Y. *et al.* Simultaneous removal of ammonium ions and sulfamethoxazole by ozone regenerated high silica zeolites. *Water Res* **188**, 116472, doi:10.1016/j.watres.2020.116472 (2021).

6 Chen, Q., Zhou, K., Chen, Y., Wang, A. & Liu, F. A novel poly ligand exchanger - Cu(II)-loaded chelating resin for the removal of ammonia-nitrogen in aqueous solutions. *Environ. Technol.* **38**, 2824-2834, doi:10.1080/09593330.2017.1278793 (2017).
